# Biodistribution and dosimetry of the GluN2B-specific NMDA receptor PET radioligand (R)-[^11^C]Me-NB1

**DOI:** 10.1186/s13550-022-00925-8

**Published:** 2022-08-26

**Authors:** Lucas Rischka, Matej Murgaš, Verena Pichler, Chrysoula Vraka, Ivo Rausch, Dietmar Winkler, Lukas Nics, Sazan Rasul, Leo Robert Silberbauer, Murray Bruce Reed, Godber Mathis Godbersen, Jakob Unterholzner, Patricia Handschuh, Gregor Gryglewski, Thomas Mindt, Markus Mitterhauser, Andreas Hahn, Simon Mensah Ametamey, Wolfgang Wadsak, Rupert Lanzenberger, Marcus Hacker

**Affiliations:** 1 Department of Psychiatry and Psychotherapy, Comprehensive Center for Clinical Neurosciences and Mental Health (C3NMH), Medical University of Vienna, Waehringer Guertel 18-20, 1090 Vienna, Austria; 2 Department of Biomedical Imaging and Image-Guided Therapy, Division of Nuclear Medicine, Medical University of Vienna, Waehringer Guertel 18-20, 1090 Vienna, Austria; 3 Department of Pharmaceutical Sciences, Division of Pharmaceutical Chemistry, University of Vienna, Vienna, Austria; 4 Center for Medical Physics and Biomedical Engineering, Medical University of Vienna, Vienna, Austria; 5 Center for Radiopharmaceutical Sciences ETH-PSI-USZ, Institute of Pharmaceutical Sciences ETH, Zurich, Switzerland; 6 Ludwig Boltzmann Institute Applied Diagnostics, Vienna, Austria; 7 Institute of Inorganic Chemistry, Faculty of Chemistry, University of Vienna, Vienna, Austria; 8 Center for Biomarker Research in Medicine (CBmed), Graz, Austria

**Keywords:** GluN2B-subunit, NMDA receptor, PET, Dosimetry, Biodistribution, Neuropsychiatric disorders

## Abstract

**Background:**

The NMDA receptor (NMDAR) plays a key role in the central nervous system, e.g., for synaptic transmission. While synaptic NMDARs are thought to have protective characteristics, activation of extrasynaptic NMDARs might trigger excitotoxic processes linked to neuropsychiatric disorders. Since extrasynaptic NMDARs are typically GluN2B-enriched, the subunit is an interesting target for drug development and treatment monitoring. Recently, the novel GluN2B-specific PET radioligand *(R)*-[^11^C]Me-NB1 was investigated in rodents and for the first time successfully translated to humans. To assess whether *(R)*-[^11^C]Me-NB1 is a valuable radioligand for (repeated) clinical applications, we evaluated its safety, biodistribution and dosimetry.

**Methods:**

Four healthy subjects (two females, two males) underwent one whole-body PET/MR measurement lasting for more than 120 min. The GluN2B-specific radioligand *(R)*-[^11^C]Me-NB1 was administered simultaneously with the PET start. Subjects were measured in nine passes and six bed positions from head to mid-thigh. Regions of interest was anatomically defined for the brain, thyroid, lungs, heart wall, spleen, stomach contents, pancreas, liver, kidneys, bone marrow and urinary bladder contents, using both PET and MR images. Time-integrated activity coefficients were estimated to calculate organ equivalent dose coefficients and the effective dose coefficient. Additionally, standardized uptake values (SUV) were computed to visualize the biodistribution.

**Results:**

Administration of the radioligand was safe without adverse events. The organs with the highest uptake were the urinary bladder, spleen and pancreas. Organ equivalent dose coefficients were higher in female in almost all organs, except for the urinary bladder of male. The effective dose coefficient was 6.0 µSv/MBq.

**Conclusion:**

The GluN2B-specific radioligand *(R)*-[^11^C]Me-NB1 was well-tolerated without reported side effects. Effective dose was estimated to 1.8 mSv when using 300 MBq of presented radioligand. The critical organ was the urinary bladder. Due to the low effective dose coefficient of this radioligand, longitudinal studies for drug development and treatment monitoring of neuropsychiatric disorders including neurodegenerative diseases are possible.

*Trial registration* Registered on 11th of June 2019 at https://www.basg.gv.at (EudraCT: 2018-002933-39).

**Supplementary Information:**

The online version contains supplementary material available at 10.1186/s13550-022-00925-8.

## Background

The *N-*Methyl-D-aspartate receptor (NMDAR) is a glutamate-gated ion channel involved in physiological processes in synaptic transmission [1]. NMDARs form heterotetrameric complexes comprising mostly two GluN1 and two GluN2 (2A–2D) subunits [2]. Moreover, NMDARs can be divided in synaptic and extrasynaptic receptors. While synaptic NMDAR are linked to protective processes, activated extrasynaptic NMDAR, typically enriched with the GluN2B-subunit, might trigger excitotoxic mechanisms. These are thought to be related to neuropsychiatric disorders such as Alzheimer’s disease or depression [3]. Hence, the GluN2B-subunit might be a promising target for drug development and to identify alterations in neuropsychiatric disorders. Unfortunately, GluN2B-specific antagonists such as EVT-101 or CERC-301 only showed results in vitro and had limited effects in clinical studies [4]. The promising GluN2B-selective antagonist CP-101,606 demonstrated similar effects to the anesthetic and rapid-acting antidepressant ketamine but subjects suffered from adverse side effects leading to discontinuation of the study [5]. The possibility to map GluN2B-subunits with and without such antagonists would pave the way to develop novel drugs with a higher efficacy by elucidating their mechanisms of action.

Positron emission tomography (PET) is an important tool in clinical routine to identify tumors or aid the diagnostic process of neuropsychiatric disorders. Moreover, PET is crucial for researching and mapping receptors or to improve drug development by highlighting their modes of action. Recently, the novel GluN2B-subunit NMDAR-specific radioligand *(R)*-[^11^C]Me-NB1 was developed in rodents [6] and successfully translated to humans [7] with a high specificity, good test--retest reliability and favorable kinetics. However, it is not only important that the tracer can penetrate the blood–brain barrier and shows promising clinical characteristics, it further has to be safe for human applications in terms of tolerability, with reasonable biodistribution and limited radiation burden. This would enable longitudinal studies with the opportunity to assess interventional effects. Therefore, we investigated the biodistribution, the equivalent organ absorbed dose coefficient and effective dose coefficient of the promising GluN2B-subunit NMDAR-specific radioligand *(R)*-[^11^C]Me-NB1.

## Methods

### Participants

Four healthy subjects (see Table 1) were recruited for the study. All participants were free from internal, neurological or psychiatric disorders assessed via a thorough medical history, physical examination, electrocardiogram and routine laboratory parameters. Exclusion criteria included neurological diseases or psychiatric disorders, illness 2 weeks prior to recruitment, history of drug or atopic allergy, myocardial infarction, history of cancer, liver or renal disease, family history of prolonged QT-interval, MR or PET contraindications, consumption of tobacco products 3 months before recruitment, history of drug or alcohol abuse, significant prior radiation exposure in the past 10 years and for females breast feeding or pregnancy. Female subjects underwent a urinary pregnancy test at the screening visit and on the measurement day. For safety reasons, vital signs of subjects were measured before and after the PET examination. Participants were observed for 3 h after the end of the scanning procedure to monitor potential adverse events.

**Table 1:** Subject information

Subject ID	Age (years)	Sex (M/F)	Weight (kg)	Height (m)	Injected dose (MBq/kg)	Total administered activity (MBq)
1	25	M	82	1.79	3.39	278.3
2	30	M	83	1.89	3.58	296.8
3	23	F	53	1.58	5.66	299.8
4	21	F	68	1.70	5.04	342.9

Demographic information describing age (years), sex (M = male, F = female), weight (kg), height (m) injected dose (MBq/kg of body weight) and total administered activity (MBq) of each subject

All subjects gave written informed consent after explanation of the study protocol. The study was approved by the Ethics Committee of the Medical University of Vienna (ethics number: 1980/2018) and registered on https://www.basg.gv.at (EudraCT: 2018-002933-39). Procedures were carried out in accordance with the Declaration of Helsinki. Subjects were insured and reimbursed for participation.

### Radiosynthesis

Syntheses were performed as previously described [6, 7]. In short, *(R)*-[^11^C]Me-NB1 was produced on a fully-automated GE Tracerlab™ FX2 C synthesis module by a methylation reaction. [^11^C]CH_3_I was reacted with the enantiomerically pure des-methyl GMP-grade precursor *(R)*-NB1 in DMF and in the presence of Cs_2_CO_3_ and separated from radiochemical impurities via semi-prep HPLC. The final product was purified using solid phase extraction, eluted with 1.5 mL ethanol and formulated for human application using 10 mL of NaCl (0.9%) and 6 mL of phosphate-buffered saline.

Molar activity was 679 ± 207 GBq/µmol at end of syntheses and 328 ± 112 GBq/µmol at the time of application with the limit of molar activity above > 25 GBq/µmol. The limits of *(R)*-Me-NB1 and precursor NB-1 concentration were both set below < 0.01 mg/mL. Mean injected dose of the active pharmaceutical ingredient (API) *(R)*-Me-NB1 was 1.6 ± 0.7 ng/kg body weight (female 2.1 ng/kg, male 1.2 ng/kg), while the mean injected dose of tracer was 305 ± 28 MBq. Therefore, the applied concentration was far below the application limits and subsequently, no pharmacokinetic effects are expected or were observed.

### Positron emission tomography

All participants underwent one whole-body PET measurement from head to mid-thighs on a fully-integrated PET/MR scanner (Siemens mMR Biograph, Erlangen, Germany). PET data were acquired in nine passes of increasing duration (0.5, 0.5, 0.5, 1, 1, 2, 3, 4 and 7 min per bed position) in six bed positions, yielding a measurement time of more than 2 h. The radioligand *(R)*-[^11^C]Me-NB1 was administered as short bolus via a cubital vein (mean injected dose: 305 ± 28 MBq; 4.4 ± 1.1 MBq/kg body weight). Subsequently, the cannula was flushed with saline. Subjects were instructed not to fall asleep and to let their thoughts wander.

### Magnetic resonance imaging

During all nine passes, a two-point DIXON sequence (DIXON, TE/TR = 2.46/3.6 ms, flip angle = 10°, voxel size = 2.6 × 2.6 × 3.1 mm^3^) was conducted during breath hold after exhalation in each bed position yielding nine whole body DIXON attenuation correction (AC) maps [8]. Due to the short frame durations, no breathing commands were given during the first five cycles. The AC maps were segmented into bone, lung, soft and fatty tissue and air.

Additionally, in the ninth cycle, a T2-weighted MR image was acquired (TE/TR = 121/1400 ms, flip angle = 133°, voxel size = 1.5 × 1.5 × 7.2 mm^3^) in each bed position to rule out gross anatomical abnormalities.

### PET processing

The 3D list-mode PET data were reconstructed with an ordinary Poisson-ordered subset expectation maximization algorithm (OP-OSEM, 3 iterations, 21 subsets). Data were corrected for attenuation, scatter, randoms and dead time. AC of the first five passes was performed with the DIXON AC map of the sixth pass due to omitted breathing commands for the first passes. Passes six to nine were corrected with the corresponding AC map. Decay correction was performed for calculation of standardized uptake values (SUV) only. SUV was defined as

1 $${\text{SUV}} = \frac{{\text{activity concentration}}}{{\left( {{\text{injected dose}}/{\text{body weight}}} \right)}}$$

### Biodistribution and dosimetry

Regions of interest (ROIs) were manually drawn on the PET images for brain, thyroid, lungs, heart wall, liver, spleen, kidneys, stomach (fundus), pancreas, bone marrow (substituted by the vertebrae L4 and L5 [9]) and urinary bladder contents using PMOD 4.2 (PMOD Technologies LLC, Zurich, Switzerland). The bladder was delineated with respect to the volume change over the measurement time. PET visualization thresholds were set individually for each subject. The threshold was set as low as possible to visualize the organs, while ROIs were drawn to cover most of the organs, but also to leave enough space to the borders and other organs to omit partial volume and breathing artifacts. The localization of each ROI was crosschecked with the matching MR image.

To estimate the effective dose coefficient, time activity curves (TACs) were extracted for all mentioned ROIs using PMOD. The cumulative activity (kBq × h) was defined as the non-decay corrected area under the curve (*AUC*; kBq/mL × h), utilizing a trapezoid method, multiplied by the respective mass of the organ (*m*_*o*_; male or female) of the OLINDA/EXM International Commission on Radiation Protection 89 (ICRP89) phantom. As the previous integration using the trapezoid function covers more than five physical half-lives of the carbon-11, the AUC from the last time point to infinity (*AUC*_inf_) was mathematically added, assuming only physical decay, as follows:

2 $${\text{AUC}}_{{{\text{inf}}}} = \frac{{A_{{{\text{last}}}} }}{k}$$

where *A*_last_ is the activity measured in the last time point and *k* represents decay constant of carbon-11. Subsequently, the cumulative activity was normalized by the injected dose (*ID*; kBq) yielding time-integrated activity coefficients (TIAC; h). Finally, the resulting TIACs were multiplied by the factor between the subject’s weight (*m*_*s*_; kg) and the phantom weight (*m*_*p*_; 60 kg and 73 kg for females and males, respectively) and were averaged for the male and female models. The TIAC for one organ of an individual subject was calculated as follows:

3 $${\text{TIAC}} = \frac{{AUC \times m_{o} }}{ID} \times \frac{{m_{s} }}{{m_{p} }}$$

The TIACs of all mentioned ROIs were entered into OLINDA/EXM 2.1 (Hermes Medical Solution, Stockholm, Sweden) [10] for each sex to obtain organ equivalent dose coefficients (*h*; Sv/Bq) and contributions to effective dose coefficients (*e*_con_; Sv/Bq) with tissue weighting factors (*w*_*r*_) according to ICRP 103 [11]. The relationship between *e*_con_ and *h* for each organ can be then defined as:

4 $$e_{{{\text{con}}}} \left( o \right) = w_{r} \left( o \right) \times h\left( o \right)$$

The TIAC of the total remainder of the body was estimated as the theoretical TIAC for carbon-11 (*T*_*1/2*_/ln(2) = 0.49 h, with *T*_*1/2*_ being the physical half-life of the radionuclide [12]) minus the sum of the regional TIACs.

5 $${\text{TIAC}}_{{{\text{remainder}}}} = \frac{{T_{1/2} }}{{{\text{ln}}\left( 2 \right)}} - \mathop \sum \limits_{o} {\text{TIAC}}\left( o \right)$$

No gastrointestinal transit or bladder voiding model was used for a conservative estimation of the effective dose (*E*; Sv). In a final step, the effective dose coefficient (*e*; Sv/Bq) was calculated as the averaged sum over the organs

6 $$e = \mathop \sum \limits_{o} \frac{{e_{{{\text{con}}}} \left( o \right)^{{{\text{male}}}} + e_{{{\text{con}}}} \left( o \right)^{{{\text{female}}}} }}{2}$$

## Results

### Safety

The GluN2B-specific radioligand *(R)*-[^11^C]Me-NB1 was well-tolerated by all subjects, and no side effects or adverse events were reported during the measurement or the subsequent 3-h time interval. The limit for the API in the clinical PET studies were set at a level of 10 µg/mL (overall max. 0.175 mg/production batch). Based on animal toxicity studies, the administered mean injected dose (1.6 ± 0.7 ng/kg body weight) was 62,500-fold lower than the no-observed-adverse-effect level (NOAEL) for *(R)*-Me-NB1. Vital signs were comparable prior and after the measurement. Furthermore, all subjects successfully passed the final screening visit.

### Biodistribution

**Fig. 1:**
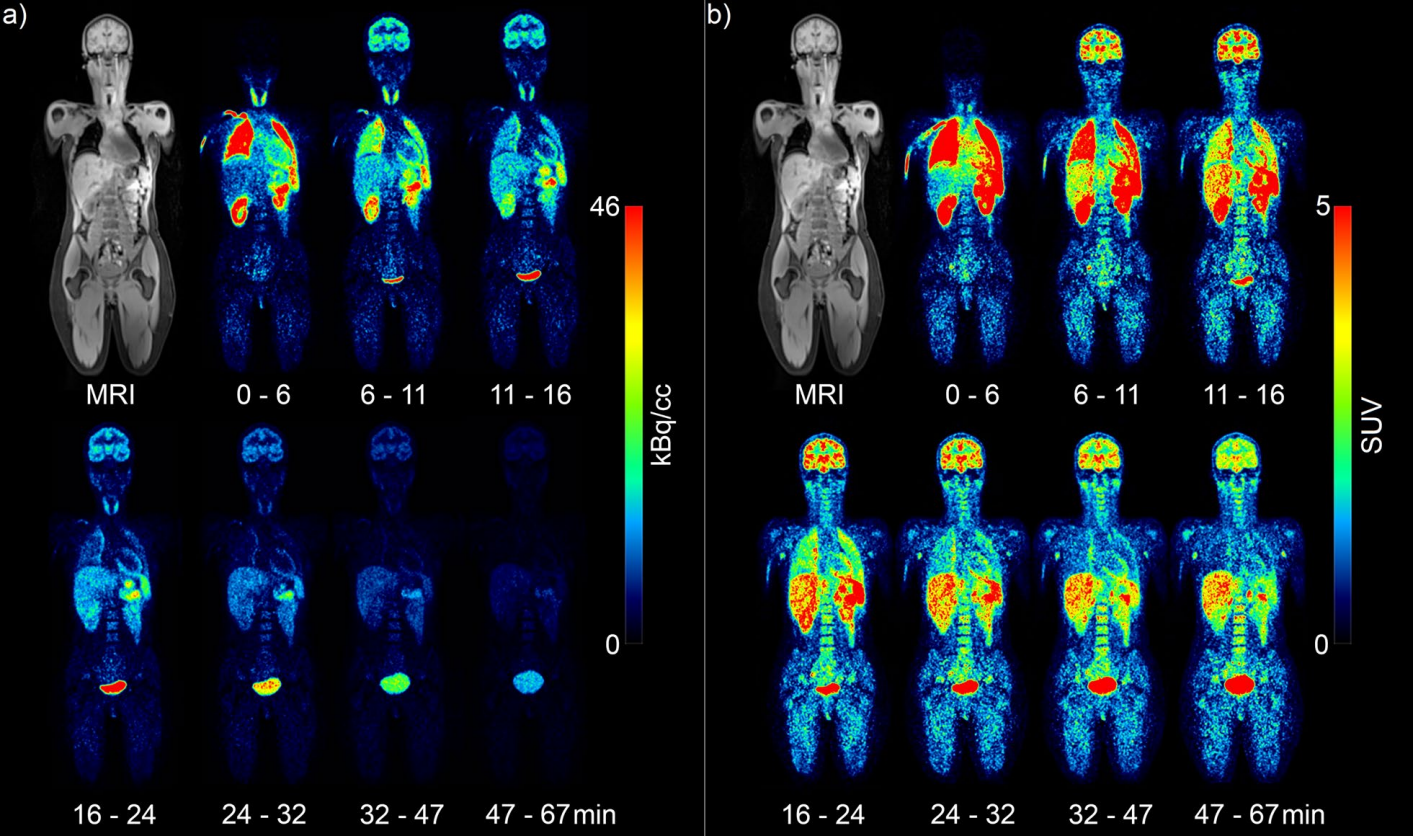
Tracer distribution over time of one representative subject. Depicted is an MR image for anatomical comparison to the PET images: **a** activity in kBq/cc (not decay corrected); **b** standardized uptake value (SUV; decay corrected). The radioligand *(R)*-[^11^C]Me-NB1 showed high initial uptake in the thyroid, lungs, spleen, pancreas and kidneys. In contrast, activity in the bone marrow (substituted by the vertebrae L4 and L5) and the liver built up over a longer period. Increase in the bladder volume over time is clearly visible. The first seven cycles are shown because of the advanced time and the low activity in the last cycles

**Fig. 2:**
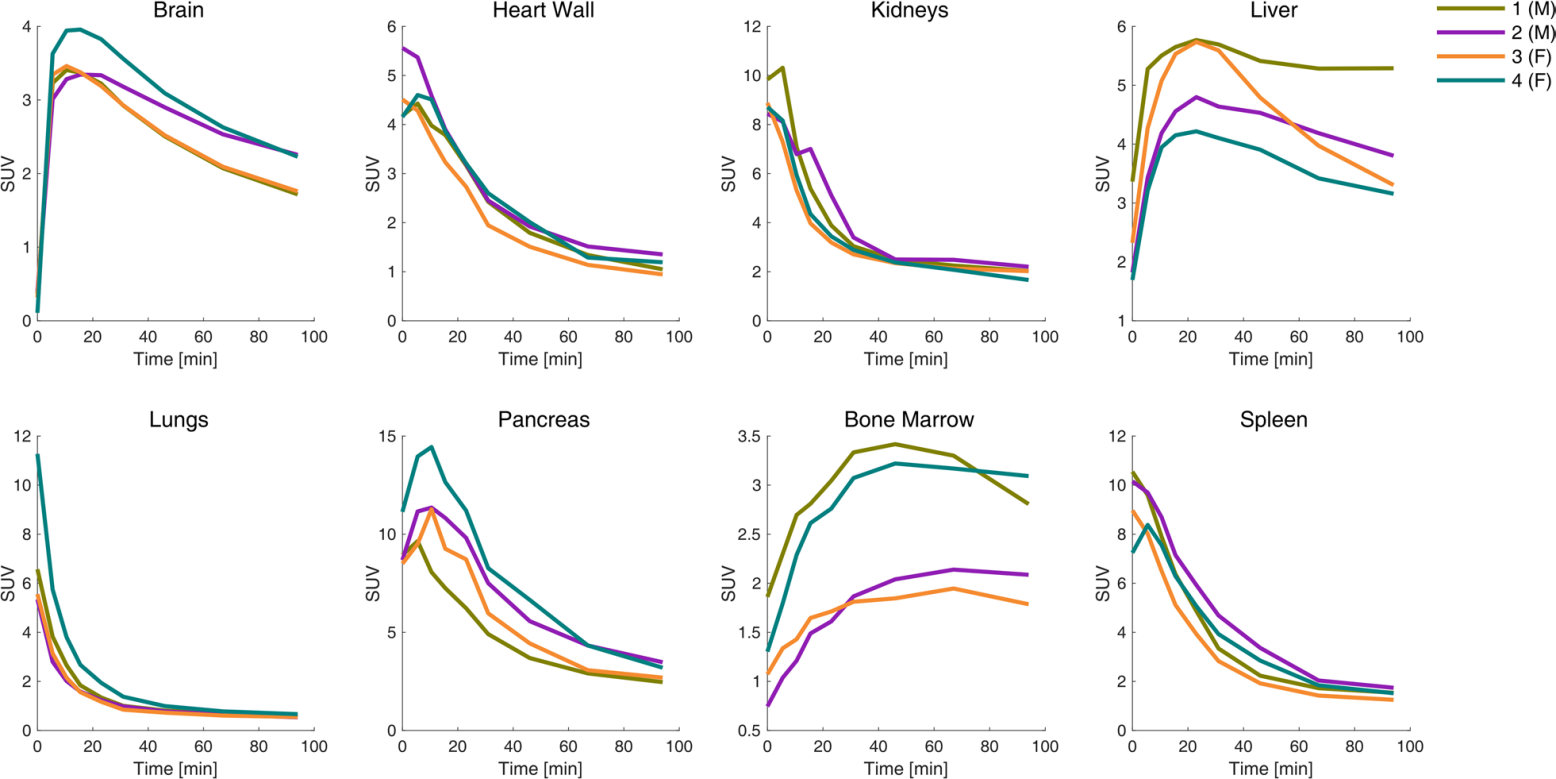
Standardized uptake values of all subjects across time (decay corrected). In the heart wall, kidneys and lungs a fast washout can be observed. Brain, liver and especially bone marrow show slower kinetics

*(R)*-[^11^C]Me-NB1 biodistribution is shown in Fig. 1. A video of the activity change per pass can be found in Online Resource 1 (not decay corrected) (Additional file 1). Initial high activity was found in the thyroid, lungs, spleen, kidneys and pancreas. While most of the mentioned regions showed a quick activity washout, the pancreas indicated clearance at a later time point of the measurement. The volume and activity concentration change in the urinary bladder is clearly visible over the measurement period. Importantly, the radioligand passed the blood–brain barrier within the first minutes. SUV TACs of all subjects are shown in Fig. 2. The radioligand was quickly washed out from the heart wall, lungs, spleen and the kidneys. Slower kinetics were observed in the liver and the brain. Interestingly, the bone marrow almost reached a steady state over the course of the 2-h measurement. The highest SUV by far was found in the urinary bladder, followed by the pancreas and spleen. There was no visual SUV difference between male and female subjects.

### Dosimetry

The TIACs of all delineated ROIs are given in Table 2. The highest TIAC was found in the urinary bladder content of one male subject with an at least 4 times higher value than in the same region of the other subjects. Other ROIs with high TIACs included the liver, brain and bone marrow. In Table 3, the equivalent dose coefficient and effective dose coefficient calculated with OLINDA/EXM are shown. Since the tissue radiation weighting factor *w*_*r*_ for electrons and photons for gamma and beta radiation is equal to 1, equivalent dose coefficients calculated with OLINDA/EXM are the same as individual organ an absorbed dose coefficients. Contribution to effective dose coefficient was the highest in the urinary bladder wall (1.5 µSv/MBq, one male subject with at least threefold higher dose compared to the other subjects) followed by the stomach wall (1.0 µSv/MBq) and the lungs (0.7 µSv/MBq). The regions with the lowest doses for all subjects included the salivary glands, thymus and small intestine. The effective dose coefficient was estimated at 6.0 µSv/MBq.

**Table 2:** Time-integrated activity coefficients in hours (h)

Organs	mean (male)	sd (male)	mean (female)	sd (female)
Brain	2.68E-02	2.22E-04	3.15E-02	1.98E-03
Heart wall	6.62E-03	3.12E-04	6.93E-03	2.38E-04
Kidneys	1.02E-02	1.08E-03	1.14E-02	7.70E-04
Liver	6.16E-02	4.17E-03	4.37E-02	2.42E-03
Lungs	1.64E-02	1.19E-03	2.05E-02	6.18E-03
Pancreas	6.58E-03	5.11E-04	9.24E-03	8.52E-04
Bone marrow	1.79E-02	5.03E-03	1.50E-02	3.73E-03
Spleen	5.25E-03	4.81E-04	6.23E-03	5.10E-04
Stomach	5.58E-03	3.02E-03	1.08E-02	1.50E-03
Thyroid	4.56E-04	1.08E-04	5.21E-04	4.50E-05
Urinary bladder	7.54E-02	5.84E-02	2.43E-02	3.98E-03
Total remainder	2.57E-01	6.74E-02	3.10E-01	3.37E-03

Average time-integrated activity coefficients in hours (*TIAC*; h) of all delineated ROIs of male and female subjects

**Table 3:** Organ equivalent doses and contribution to effective doses coefficient

Organs	h (male) (µSv/MBq)	h (female) (µSv/MBq)
Adrenals	4.4	5.9
Brain	6.2	8.1
Breasts	–	2.4
Esophagus	2.7	3.6
Gallbladder Wall	4.3	4.1
Left colon	2.8	3.6
Small Intestine	2.9	3.4
Stomach Wall	6.1	10.5
Right colon	2.8	3.4
Rectum	3.7	4.1
Heart Wall	6.9	9.1
Kidneys	10.5	12.9
Liver	11.9	11.3
Lungs	4.7	7.0
Ovaries	–	3.5
Pancreas	14.3	22.6
Prostate	4.5	–
Salivary Gland	2.1	2.8
Bone Marrow	3.9	4.6
Osteogenic Cells	3.1	3.4
Spleen	10.5	14.6
Testes	2.2	–
Thymus	2.3	3.4
Thyroid	6.3	8.3
Urin. Bladder Wall	53.6	22.3
Uterus	–	4.2
Remainder of Body	2.7	3.5
Effective Dose coefficient (µSv/MBq)	6.0	

The organ equivalent dose coefficient *h* in µSv/MBq of each organ and the effective dose coefficient in µSv/MBq were calculated with OLINDA/EXM

## Discussion

In this study, we evaluated the safety, biodistribution and dosimetry of the promising GluN2B-specific NMDAR radioligand *(R)*-[^11^C]Me-NB1 in humans. The radioligand was well-tolerated by all subjects and no adverse events were reported. The effective dose coefficient was 6.0 µSv/MBq with the urinary bladder as the critical organ (contribution to effective dose coefficient: 1.5 µSv/MBq). Of note, the no bladder voiding model was applied for conservative estimation of effective dose coefficient, as PET measurement durations of 120 min (i.e., approx. six half-lives) are common.

The calculated effective dose coefficient of 6.0 µSv/MBq is similar to other carbon-11 tracers such as [^11^C]Raclopride [13], [^11^C]FLB 457 [14] or [^11^C]PE2I [15]. In comparison with other PET radioisotopes, it is lower than [^64^Cu]DOTA-AE105 (31.5 µSv/MBq) [16] or [^18^F]FDG (19.0 µSv/MBq) [17]. On the other hand, the effective dose coefficient of *(R)*-[^11^C]Me-NB1 is higher than that of [^15^O]water (0.9 µSv/MBq) [18]. Usually, sex differences are observed with a higher equivalent dose coefficient in females than in males. This might be caused by a smaller model in the OLINDA/EXM software where organs are closer together and therefore, irradiating each other to a higher degree than in the male model [12]. Another cause could be the additional radiation exposure of breasts and uterus [19]. Also in this study sex differences were found in most of the regions but not in the equivalent dose coefficient. A reason might be the high urinary bladder uptake of the first male subject. Whether this value was an outlier or valid remains unknown because of the small sample size.

Interestingly, Zanotti-Fregonara et al. recently discussed the necessity of carbon-11 dosimetry studies in general [20]. The effective dose coefficient relies on the applied methodology, including if a bladder voiding model was used. Comparing the effective dose coefficient of the same carbon-11 tracers in two or more studies, performed by different groups, revealed an average difference of more than 40% in the majority of the studies. However, the variability in the effective dose coefficient across all investigated carbon-11 dosimetry studies was small. In consequence, the authors stated dosimetry studies with such short-lived radionuclides may be unnecessary in the future, both in animals and humans and radiation exposure could be avoided. Instead, it was proposed that a value of 5.0 µSv/MBq should be applied to studies using carbon-11 tracers, which was the average across all investigated studies [20]. We support this notion to protect animals and humans from potentially harmful and unnecessary irradiation. In addition, avoiding dosimetry studies of carbon-11 tracers, would decrease the financial burden and avoid the use of the resources of the institution.

Applying the effective dose coefficient found in this study (i.e., 6.0 µSv/MBq) would yield a dose of 2.7 mSv for a 75 kg person and an administered dose of 6.0 MBq/kg [7]. According to the Austrian Medical Radiation Protection law, subjects are allowed to receive a dose of 30.0 mSv within 10 years, excluding medically indicated examinations or studies with a benefit for the individual. In addition, organ absorbed doses presented in this study are all below the threshold doses for the occurrence of deterministic radiation effects. In the European Union, individual regulations apply. Under the regulations of the Radioactive Drug Research Committee (RDRC) applied in the USA, doses are limited up to 50.0 mSv per single dose and 150.0 mSv per year, depending on the organ. Differently, the ICRP recommends a yearly effective dose of < 10.0 mSv. In any of the mentioned regulations, longitudinal studies with *(R)*-[^11^C]Me-NB1 are feasible with several measurements per year, enabling drug development and interventional studies. Moreover, during PET/CT measurements, CT contributes with a significant portion of the radiation dose hence using MR imaging instead of classical CT helps to keep the radiation dose as reasonably low as possible. Alternatively, one could consider use of the low-dose CT to prevent an extra radiation burden to measured subject or personnel.

Although the effective dose coefficient of 6.0 µSv/MBq was similar to other studies, we have to mention a few limitations. The sample size was relatively small and only young adults were recruited. Another issue might be the use of a PET/MR with MR-based AC. While the MR has the advantage of no additional irradiation and a higher contrast for soft tissue, the MR-based AC method DIXON is known to underestimate activity. However, we used a method extending the AC map with bone models to mitigate this effect [8]. Finally, the ninth pass lasted more than 7 min in each bed position (total pass > 42 min). Yet, the second to sixth bed positions were decay corrected to the first, assuming only physical decay. Nonetheless, at the ninth pass, approximately more than five half-lives had already passed and little activity was left (see seventh cycle of Fig. 1).

## Conclusion

The Glu2NB-subunit NMDAR-specific radioligand *(R)*-[^11^C]Me-NB1 is safe to be applied in clinical studies. The dosimetry study revealed an effective dose coefficient of 6.0 µSv/MBq with the critical organ being the urinary bladder. Considering a reasonable dose of 500 MBq, this would yield an effective dose of 3.0 mSv. Thus, the radioligand enables longitudinal studies investigating the GluN2B-subunit NMDAR for drug development, interventional studies and alterations in neuropsychiatric disorders including neurodegenerative diseases under the commonly applied regulations of the RDRC and the ICRP guidelines.

## Supplementary Information

**Additional file 1**. A video showing the time change of activity distribution in whole body (not decay corrected).

## Data Availability

Raw data are not available due to reasons of data protection. Processed data are available from the corresponding author on reasonable request and a data-sharing agreement.

## Abbreviations

AC: Attenuation correction
API: Active pharmaceutical ingredient
AUC: Area under the curve
CT: Computed tomography
ICRP89: International commission on radiation protection 89
NMDAR: NMDA receptor
NOAEL: No-observed- adverse-effect level
OP-OSEM: Ordinary Poisson-ordered subset expectation maximization algorithm
PET: Positron emission tomography
RDRC: Radioactive drug research committee
ROI: Regions of interest
SUV: Standardized uptake values
TAC: Time activity curves
TIAC: Time-integrated activity coefficient
